# Concordance with final pathology when transitioning from standard transrectal to cognitive targeted transperineal prostate biopsy

**DOI:** 10.1002/bco2.486

**Published:** 2025-01-14

**Authors:** Alfred Honoré, Karsten Gravdal, Patrick Juliebø‐Jones, Lars Anders Rokne Reisæter, Christian Beisland, Christian Arvei Moen

**Affiliations:** ^1^ Department of Urology Haukeland University Hospital Bergen Norway; ^2^ Department of Clinical Medicine University of Bergen Bergen Norway; ^3^ Department of Pathology Haukeland University Hospital Bergen Norway; ^4^ Department of Radiology Haukeland University Hospital Bergen Norway

**Keywords:** biopsy, cognitive fusion, concordance, pathology, prostate cancer, RARP, surgery outcomes, transperineal biopsy

## Abstract

**Objective:**

Transrectal (TR) prostate biopsy is being increasingly abandoned in favour of a transperineal (TP) approach as well as a targeted biopsy only of the index lesion(s). It remains underreported how these changes could impact concordance at final pathology. We aimed to evaluate the impact of transitioning from standard transrectal (sTR) to cognitive targeted transperineal (cog‐tTP) biopsy on final pathology including concordance and upgrading.

**Material and methods:**

Analysis of consecutive patients undergoing prostate biopsy and prostatectomy (RP) between January 2018 and May 2022 at a tertiary centre in Western Norway.

**Results:**

There were 210 and 239 patients in the sTR and cog‐tTP groups, respectively. The mean [IQR] number of biopsies decreased from 12 [4–12] to 3 [3–4] (*p* < 0.001). The overall rate of concordance between biopsy and final pathology was 64% in both groups (Table 3, Figure 1). 24% Twenty‐four per cent (cog‐tTP) versus 19% (sTR) had grade group (GG) upgrading, while 12% versus 17% were downgraded (*p* = 0.2). Regarding positive surgical margins (PSMs) that were >3 mm in extension, there were only 3.3% and 2.1% in the sTR and cog‐tTP groups, respectively (*p* = 0.4). For surgical outcomes associated with RP, no differences in terms of postoperative complications between the groups were found (cog‐tTP:10% vs. sTR:6%, *p* = 0.10).

**Conclusion:**

Transitioning from sTR biopsy to targeted cog‐tTP biopsy does not compromise concordance at final pathology nor does it increase the risk of tumour upgrading.

## 1 INTRODUCTION

Recent years have witnessed the increased uptake of transperineal (TP) biopsies to diagnose prostate cancer (PCa) as well the abandonment of transrectal (TR) biopsies across many centres worldwide. Indeed, this transition is recommended by the European Association of Urology (EAU) despite the acknowledged logistical challenges associated with it.^1^ This evolution of clinical practice has been largely driven by a body of evidence, which has revealed a reduction in infectious complications associate with TP biopsies, which obviates the need for routine antibiotic prophylaxis. This holds the potential for clear advantages in terms of antibiotic stewardship. However, while the complication burden is an important aspect to consider when comparing these biopsy methods, so too is diagnostic accuracy. One means to evaluate this is in terms of concordance at final pathology as examined on the radical prostatectomy specimen.

Another modification that has gained momentum is the adoption of a targeted biopsy (TB) strategy, combined with the transperineal (TP) approach.^2^, ^3^, ^4^, ^5^ This can be performed using cognitive and/or software fusion methods. Debate is ongoing regarding which method is superior. Proponents of the software fusion method argue that the burden of operator dependence associated with a cognitive approach increases the risk of human error. Further studies exploring whether adopting cognitive targeted biopsy as a routine approach are therefore needed.

The aim was to evaluate the impact of transitioning from mainly standard transrectal (sTR) to cognitive targeted transperineal (cog‐tTP) biopsy after making a full transition from the former to the latter approach at our centre. The focus of this evaluation was the concordance rates at final pathology and the risk for tumour upgrading.

## 2 MATERIAL AND METHODS

### 2.1 Patient selection and data collection

As part of an ethically approved study (REK 2022‐465105), patients who underwent robot‐assisted radical prostatectomy (RARP) were reviewed and compared by diagnostic approach. The study time period was between 1 January 2018 and 31 May 2022, and all cases took place at Haukeland University Hospital, a tertiary centre in Western Norway. In January 2020, full implementation of (cog‐tTP) biopsy as the routine method for prostate biopsy at our centre took place. A step‐by‐step description of the coaxial freehand technique including further logistic details of this transition has been published previously.^6^ Of note, transperineal biopsies were performed using local anaesthesia only as the routine method over the whole‐time period. All biopsy approaches and strategies (standard and cognitive targeted) were eligible for inclusion as well as biopsies performed for either initial diagnosis or as part of active surveillance (AS).

### 2.2 Outcomes of interest

The primary outcomes of interest were degree of concordance of grade group (GG) at biopsy versus final pathology between sTR and cog‐tTP groups as well as the risk of GG upgrading. Given how an increase in either positive surgical margins (PSMs) rates or surgical complications after transitioning could represent surrogates of worse diagnostic accuracy, these were selected as secondary outcomes of interest.

### 2.3 Diagnostic evaluation

MRI scans were performed using a 1.5‐T or 3‐T scanner without an endorectal probe. Images were reported according to the Prostate Imaging Reporting and Data System (PI‐RADS) v2 and later, the PI‐RADS 2.1 system.^7^, ^8^ In the cog‐tTP group, three biopsies were taken of the leading lesion (PI‐RADS 4‐5) as identified on MRI. If there were two equivocal PIRAD 4+ lesions, both were biopsied. If a third lesion was seen, it would not be routinely biopsied. Unilateral PI‐RADS 3 lesions and diffuse PI‐RADS 3 changes were biopsied if the PSA‐density was >0.20 ng/mL/mL. If the PSA‐density was <0.20 ng/mL/mL and PI‐RADS was 3, biopsy was omitted. PI‐RADs 1‐2 were not biopsied in the cog‐tTP group irrespective of PSA‐density. Hitachi Preirus ultrasound machine (Hitachi Medical Corporation, Tokyo, Japan) was used for all biopsies using either an end‐fire (sTR) or linear biplane probe (cog‐tTP). Data were also collected on patient characteristics, clinical and radiological T‐stage, number of positive cores and total number of biopsy cores taken. Biopsy results were described using the International Standard of Urological Pathology (ISUP) from 2019.^9^

### 2.4 Postoperative evaluation and final pathology

Pathological GG, T‐ and N‐stage were also described according to the 2019 ISUP consensus, including PSM status.^9^ Complications were categorised according to the Clavien–Dindo system.^10^ A possible nerve‐sparing approach was planned at the multidisciplinary team (MDT) meeting based on biopsy results and MRI‐findings, and an actual nerve‐sparing procedure (uni‐ or bilateral) was confirmed in the medical records.

### 2.5 Statistical analysis

Continuous variables were reported using median and interquartile range. Categorical variables were reported using frequencies and proportions. All analyses and plots were carried out using the R4.2.2 build (R Foundation for Statistical Computing, Vienna, Austria). The Wilcoxon rank‐sum test and Pearson's 𝝌^2^‐test were used to compare groups. Tests were considered significant when *p* < 0.05.

## 3 RESULTS

### 3.1 Patient cohort

Overall, 449 patients were included in the study (sTR:210, cog‐tTP:239). The median (interquartile range, IQR) age in the TR group was lower than in the TP group (64 [60–69] vs. 66 [62–69] years, *p* = 0.027). However, there were otherwise no differences among baseline parameters including ASA, ECOG, Charlson Comorbidity Index, PSA, Prostate Volume, PSA‐density or Primary versus Surveillance biopsy (Table 1).

**TABLE 1:** Summary of patient characteristics.

Characteristic	Transperineal, *N* = 239^a^	Transrectal, *N* = 210^a^	*p*‐Value^b^
Age at biopsy	66.0 (62.0, 69.0)	64.0 (60.0, 69.0)	0.027
ECOG			>0.9
0	237 (99%)	209 (100%)	
1+	2 (0.8%)	1 (0.5%)	
ASA			>0.9
1–2	238 (100%)	209 (100%)	
3+	1 (0.4%)	1 (0.5%)	
Charlson Comorbidity Index (age adjusted)			0.3
0	3 (1.2%)	2 (0.9%)	
1	35 (15%)	38 (18%)	
2	126 (53%)	104 (50%)	
3	70 (29%)	55 (26%)	
4+	5 (2.1%)	11 (5.2%)	
PSA	8 (6, 12)	8 (6, 12)	>0.9
Prostate volume	34 (26, 47)	36 (29, 50)	0.045
PSA‐density	0.25 (0.16, 0.38)	0.22 (0.15, 0.35)	0.2
Primary biopsy vs. surveillance	178 (74%)	149 (71%)	0.4

Abbreviations: ASA, American Society of Anaesthesiology grade; BMI, Body Mass Index; ECOG, Eastern Conglomerate Oncology Group performance status; PSA, Prostate Specific Antigen.

^a^ Median (IQR); *n* (%).

^b^ Wilcoxon rank‐sum test; Fisher's exact test; Pearson's Chi‐squared test.

### 3.2 Preoperative characteristics

The number of biopsy cores was significantly lower in the cog‐tTP group, with a median (IQR) of 3 (3–4) vs 12 (4–12). Positive cores were significantly higher in the sTR group, with a median (IQR) of 4 (2–5) versus 3 (3–3). Most biopsies were primary (sTR: 71% and cog‐tTP: 74%, *p* = 0.5), with no difference between the proportions in the groups. There were no significant differences in clinical or radiological T‐stage, PI‐RADS score or biopsy GG (Table 2).

**TABLE 2:** Preoperative characteristics.

Characteristic	Transperineal, *N* = 239^a^	Transrectal, *N* = 210^a^	*p*‐Value^b^
Clinical T‐stage			0.14
1	162 (68%)	124 (59%)	
2	65 (27%)	70 (33%)	
3	12 (5.0%)	16 (7.6%)	
Radiological T‐stage			0.4
T1c	6 (2.5%)	7 (3.4%)	
T2a/b	127 (53%)	92 (44%)	
T2c	54 (22%)	55 (27%)	
T3a/b	52 (22%)	52 (25%)	
PIRAD (v2.0)			0.10
2	0 (0%)	3 (1.4%)	
3	29 (12%)	33 (16%)	
4	119 (50%)	106 (52%)	
5	91 (38%)	64 (31%)	
Biopsy cores	3.0 (3.0, 4.0)	12.0 (4.0, 12.0)	<0.001
Positive cores	3.0 (3.0, 3.0)	4.0 (2.0, 5.0)	<0.001
Biopsy ISUP grade			0.4
GG1	14 (5.9%)	16 (7.6%)	
GG2	139 (58%)	116 (55%)	
GG3	48 (20%)	45 (21%)	
GG4	16 (6.7%)	21 (10.0%)	
GG5	22 (9.2%)	12 (5.7%)	

Abbreviations: GG, Grade group; ISUP, International Society of Uropathologists; PIRAD, Prostate Imaging Reporting and Data System.

^a^ *n* (%); Median (IQR).

^b^ Pearson's Chi‐squared test; Fisher's exact test; Wilcoxon rank‐sum test.

### 3.3 Pathology results

No differences were found regarding ISUP grade, pathology stage or surgical margins (Table 3). After the transition, there was a significant reduction in extended pelvic lymph node dissections (ePLND) from 65% to 54% (*p* = 0.02). However, overall lymph node metastasis detection was not reduced (*p* > 0.9). PSMs for pT2 were 9.7% in the cog‐tTP group and 11% in the sTR group, and 10% versus 9.5% for pT3 cancers, with no statistical significance between them (*p* = 0.3). This was the case despite nerve sparing increasing from 65% (sTR) to 73% (cog‐tTP). When isolating PSMs that were >3 mm in extension, there were only 3.8% and 2% in the sTR and cog‐tTP groups, respectively (*p* = 0.4). RARP complications were similar between the groups (10% in the cog‐tTP group vs. 6% in the sTR group, *p* = 0.10).

**TABLE 3:** Postoperative characteristics.

Characteristic	Transperineal, *N* = 239^a^	Transrectal, *N* = 210^a^	*p*‐Value^b^
Pathology ISUP grade			0.3
GG1	5 (2.1%)	7 (3.3%)	
GG2	136 (57%)	130 (62%)	
GG3	47 (20%)	44 (21%)	
GG4	16 (6.7%)	7 (3.3%)	
GG5	35 (15%)	22 (10%)	
pT‐stage			0.5
T2a/b	16 (6.7%)	13 (6.2%)	
T2c	151 (63%)	144 (69%)	
T3a/b	72 (30%)	53 (25%)	
ePLND	130 (54%)	137 (65%)	0.020
LNM	20 (8.4%)	17 (8.1%)	>0.9
Nerve sparing	175 (73%)	137 (65%)	0.067
PSM			0.3
Free	192 (80%)	166 (79%)	
pT2a/b	3 (1.3%)	0 (0%)	
pT2c	20 (8.4%)	24 (11%)	
pT3a/b	24 (10.0%)	20 (9.5%)	
PSM >3 mm	6 (2.5%)	8 (3.8%)	0.4
Gleason at margin			0.2
3	38 (83%)	31 (72%)	
4+	8 (17%)	12 (28%)	
Clavien–Dindo			0.10
0	214 (90%)	198 (94%)	
1	6 (2.5%)	1 (0.5%)	
2	9 (3.8%)	5 (2.4%)	
3a	6 (2.5%)	2 (1.0%)	
3b	1 (0.4%)	4 (1.9%)	
4a	2 (0.8%)	0 (0%)	
4b	1 (0.4%)	0 (0%)	
Grade change			0.2
Downgrade	28 (12%)	35 (17%)	
Unchanged	153 (64%)	135 (64%)	
Upgrade	58 (24%)	40 (19%)	

Abbreviations: ePLND, extended pelvic lymph node dissection; GG, grade group; ISUP, International Society of Uropathologists; LNM, lymph node metastasis; PSM, Positive Surgical Margin.

^a^ *n* (%).

^b^ Pearsons Chi‐squared test; Fishers exact test.

### 3.4 Concordance and grade group migration

**FIGURE 1:**
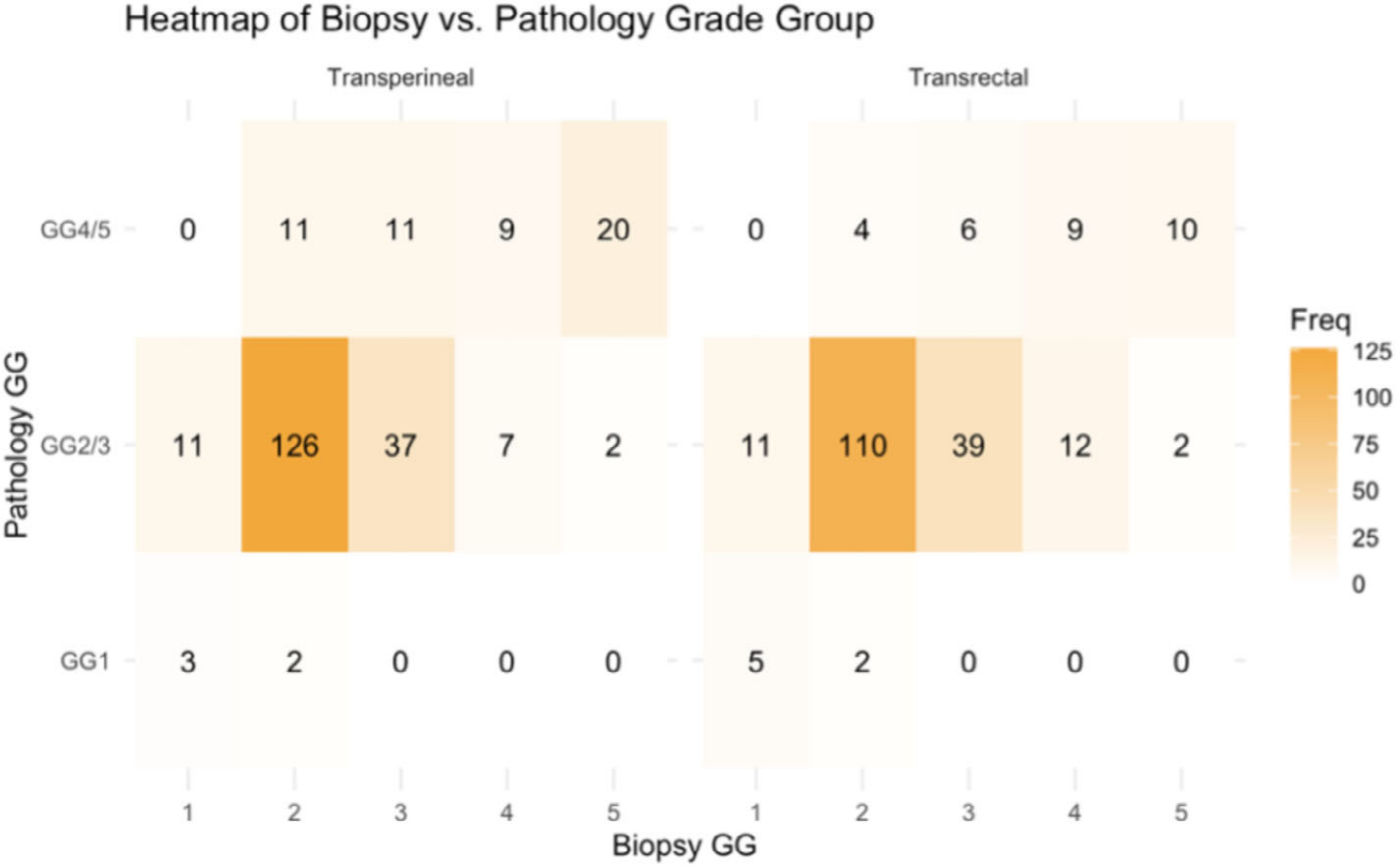
Heat map Gleason grade group at biopsy versus final pathology. Biopsy grade group (GG) is displayed horizontally, while final pathology grade group (GG) is displayed vertically. Final pathology GG is grouped by low‐, intermediate‐ and high‐risk cancer. Each cell value in the heat map represents the number of patients that fall into a specific combination of biopsy grade group and pathology grade group. Darker tones show higher values, while lighter tones show lower values. GG, grade group; GG1, low grade; GG2/3, intermediate grade; GG4/5, high grade.

The overall rate of concordance between biopsy and final pathology was 64% in both groups (Table 3, Figure 1). Twenty‐four per cent(cog‐tTP) versus 19% (sTR) were upgraded, while 12% versus 17% had GG downgrading (*p* = 0.2). There were significantly more GG4/5 in the cog‐tTP group versus the sTR group (21% vs. 14%, *p* = 0.04).

Of the 14 patients with GG1 on biopsy in the cog‐tTP group, 11 were upgraded to GG2 (11 of 16 cases were upgraded in the sTR group). These 30 patients accounted for 8% of all patients biopsied with GG1 (see previously published data^6^ and Table S1 of intention to treat for the whole cohort of patients).

**FIGURE 2:**
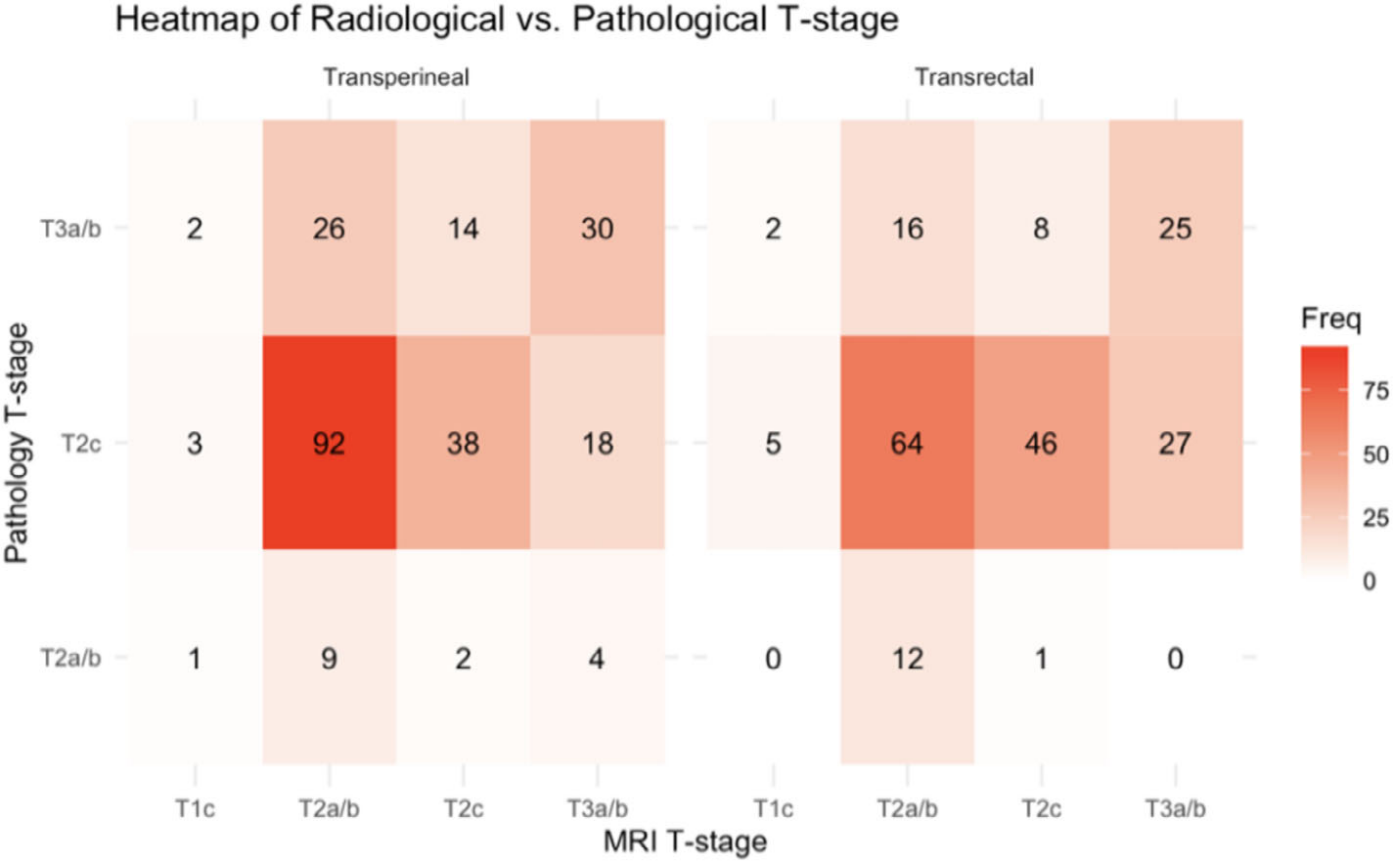
Heat map of radiological versus pathological T‐stage. MRI T‐stage is displayed horizontally, while pathology T stage is displayed vertically. Each cell value in the heat map represents the number of patients that fall into a specific combination of radiological and pathological stage. Darker tones show higher values, while lighter tones show lower values. MRI, magnetic resonance imaging; T1c, no visible lesion; T2a/b, unilateral prostate cancer; T2c, bilateral prostate cancer; T3a/b, locally advanced prostate cancer.

No significant differences in T‐stage between MRI and final pathology between cog‐tTP and sTR were seen (Figure 2). Most patients were upstaged from unilateral to bilateral (or greater) on final pathology in both groups, and only 6.2% (cog‐tTP) and 6.7% (sTR) were unilateral on final pathology.

## 4 DISCUSSION

In this real‐world example of a centre transitioning from (sTR) to cog‐tTP biopsy, oncological outcomes were not compromised in terms of concordance or tumour upgrading despite significantly fewer cores being taken. The trend towards fewer PSMs in the cog‐tTP group despite a rising number of nerve‐sparing procedures as well as the lack of significant difference in surgical complications also supports the affirmation that preoperative diagnostic accuracy has not been impeded because of this transition. There were two experienced surgeons with more than 200 RARP procedures each before 2018. As this is a teaching hospital, there have been a total of four surgeons in training throughout the study period, but none of them operated without the assistance of a more experienced surgeon until they had performed at least 50 procedures under guidance. We have also previously published our results of the learning curve and reproducibility of the cog‐tTP procedure (6). This however should not affect RARP results directly, apart from a better understanding of MRI images and lesion location when operating (all trainee surgeons in our institution are taught biopsy first before learning RARP).

The results of both the PROMIS and PRECISION studies highlight that the implementation of prebiopsy MRI helps avoid the diagnosis of low‐grade tumours while still finding the majority of significant cancers.^11^, ^12^ To this end, adopting a MRI‐TB pathway can markedly reduce the overall number of patients biopsied, the individual number of cores taken, and the amount of patients included into active surveillance.^13^

Given the association between number of cores and pain, an added advantage of a targeted approach is the potential for improved patient comfort as well as a shorter procedural time.^14^

In a recent multicentre study, Baboudjian et al. determined that MRI‐TB does not lead to a higher risk of overtreatment (17.5% downgraded). However, a software fusion approach was employed, and only 9.3% were transperineal.^15^ Our study found lower rates of downgrading in both groups (12% and 16% in the cognitive cog‐tTP and sTR groups, respectively). Therefore, cog‐tTP biopsy appears to yield similar results to software targeted biopsy.

Björklund et al. evaluated factors associated with unfavourable final pathology in a cohort of low volume GG2 PCa on biopsy. Their study revealed that >5% Gleason grade 4 on biopsy predicted a risk of upgrading to GG3+ disease.^16^ This may in part explain a larger proportion of high‐grade tumours was found in the cog‐tTP group.

Despite mainly unilateral biopsy in the cog‐tTP group, there were no significant differences in PSMs compared to the sTR group. Moreover, there was a tendency to perform more nerve‐sparing procedures in the former group. Given that >90% of the cancers were at least T2c (i.e., bilateral or locally advanced) on final pathology, one could argue that despite the EAU guidelines still recommending clinicians to combine systematic and targeted biopsy, the additional diagnostic value of sampling the contralateral side in fact appears to be minimal in the setting of radiologically unilateral cancers.^1^ Our sTR hit rate mean (IQR) of 4 (2–5) implies that 75% had ≤5 positive cores. In other words, less than half the prostate in many cases, meaning that this technique also mainly samples only the leading lesion but has more positive cores due to more biopsies taken. This is in tune with the recently updated guidelines, which suggest that perilesional/regional biopsies can be used to avoid systematic biopsies.^17^ Furthermore, performing standard biopsies may result in overdiagnosis in the context of the initial biopsy by detecting non‐significant cancers. In addition, it seems to cause the operator to omit a unilateral nerve‐sparing procedure for fear of a resultant PSM. Even if only unilateral findings of suspected PCa are identified on MRI, it is assumed that the patient may well harbour a bilateral tumour burden. However, in our study, unilateral sampling did not increase the PSM‐rate. It has been shown that PSMs are usually at the index lesion as identified on MRI.^18^ This is highly relevant when considering targeted biopsy given that biochemical recurrence is more prevalent when the PSM is >3 mm in length, and metastasis‐free survival (MFS) is related to pathology GG4/5, T3 stage and GG4+ at the PSM, not the presence of a PSM.^19^

This study is limited by its single centre status as well as comparing two groups that have changed both access type and approach and does not look separately at those who went directly to RARP versus those who were initially under AS. The groups were not randomised, which does introduce additional bias. Furthermore, both patient and treatment selection have changed over time. However, the findings represent those in a real‐world setting. Given the high volume of PCa diagnostics globally, they can potentially serve as an example for other centres considering such a transition as they reveal that it can be successfully implemented without compromising diagnostic accuracy. Future randomised studies such as the ongoing TRANSLATE trial are warranted that evaluate the diagnostic accuracy of TP versus TR as well as standard versus cognitive methods.^20^

## 5 CONCLUSION

Adopting cognitive transperineal targeted biopsy of the index lesion(s) as the routine diagnostic approach does not compromise concordance at final pathology nor is it associated with tumour upgrading.

## AUTHOR CONTRIBUTIONS

All authors made significant contributions to this work. *Design and funding*: Alfred Honore, Christian Arvei Moen and Christian Beisland. *Data collection*: Alfred Honore and Christian Arvei Moen. *Statistical analyses*: Alfred Honore and Christian Arvei Moen. *Writing the manuscript*: Alfred Honore, Karsten Gravdal, Patrick Juliebø‐Jones, Lars Anders Rokne Reisæter, Christian Beisland, and Christian Arvei Moen.

## CONFLICT OF INTEREST STATEMENT

The authors have no conflicts of interest to declare.

## Supporting information


**Table S1.** Intention to Treat. Does not include benign biopsies of the cohort. Numbers are higher for treatments than those included in this analysis since not all patients opted for the recommended treatment or were treated elsewhere. RARP – Robot Assisted Radical Prostatectomy.
