# Supplementary material for: Concordance with final pathology when transitioning from standard transrectal to cognitive targeted transperineal prostate biopsy
Source: BJUI Compass. 2025 Jan 14;6(1):e486. doi: 10.1002/bco2.486 (PMC11771486; doi:10.1002/bco2.486)
Supplement: Supplementary file 1 — Table S1. Intention to Treat. Does not include benign biopsies of the cohort. Numbers are higher for treatments than those included in this analysis since not all patients opted for the recommended treatment or were treated elsewhere. RARP – Robot Assisted Radical Prostatectomy. [file BCO2-6-e486-s001.docx]

| **Characteristic** | **Transperineal**,  N = 744^1^ | **Transrectal**,  N = 817^1^ | **p-value**^2^ |
| --- | --- | --- | --- |
| **Intention to treat** |  |  | <0.001 |
| Active Surveillance | 216 (29%) | 323 (40%) |  |
| RARP | 262 (35%) | 238 (29%) |  |
| Radiotherapy | 217 (29%) | 200 (24%) |  |
| Watchful waiting | 49 (6.6%) | 56 (6.9%) |  |
| ^1^n (%); Median (IQR) | | | |
| ^2^Pearson's Chi-squared test | | | |
